# Recent Progress and Emerging Application Areas for Lithium–Sulfur Battery Technology

**DOI:** 10.1002/ente.202000694

**Published:** 2020-11-18

**Authors:** Susanne Dörfler, Sylwia Walus, Jacob Locke, Abbas Fotouhi, Daniel J. Auger, Neda Shateri, Thomas Abendroth, Paul Härtel, Holger Althues, Stefan Kaskel

**Affiliations:** ^1^ Chemical Surface and Reaction Technology Fraunhofer IWS Dresden 01277 Germany; ^2^ OXIS Energy Ltd. Culham Science Center Abingdon UK; ^3^ TU Dresden University of Technology Dresden 01069 Germany; ^4^ Advanced Vehicle Engineering Centre Cranfield University Bedfordshire MK 43 0AL UK

**Keywords:** applications, battery management systems, lithium sulfur batteries, prototype cells

## Abstract

Electrification is progressing significantly within the present and future vehicle sectors such as large commercial vehicles (e.g., trucks and buses), high‐altitude long endurance (HALE), high‐altitude pseudosatellites (HAPS), and electric vertical take‐off and landing (eVTOL). The battery systems’ performance requirements differ across these applications in terms of power, cycle life, system cost, etc. However, the need for high gravimetric energy density, 400 Wh kg^−1^ and beyond, is common across them all, as it enables vehicles to achieve extended range, a longer mission duration, lighter weight, or increased payload. The system‐level requirements of these emerging applications are broken down into the component‐level developments required to integrate Li–S technology as the power system of choice. To adapt batteries’ properties, such as energy and power density, to the respective application, the academic research community has a key role to play in component‐level development. However, materials and component research must be conducted within the context of a viable Li–S cell system. Herein, the key performance benefits, limitations, modeling, and recent progress of the Li–S battery technology and its adaption toward real‐world application are discussed.

## Graphical abstract

The advantages, limitations, and progress of Li–S batteries are described. Approaches to extend cycle life, energy, power, and safety are further discussed. Recent advances regarding modelling and battery management of these batteries are summarized, and the requirements for as well as the integration of Li–S prototype cells in present and future real world applications is elaborated.

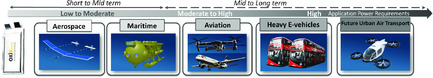

## 1 Introduction

With the ever‐increasing need for electrification across many application sectors, the development of new energy‐storage technologies is of increasing relevance and critical importance. Electrification is progressing significantly within the traditional transportation sectors such as electric bikes, cars, buses, and other commercial vehicles, enabled by continued cell development and Gigafactory‐scale mass production of Li‐ion battery (LIB) technology. However, two key factors are starting to drive the need for new solutions to be found. One factor is the secure supply of key elements—mainly cobalt and nickel—used in most of the conventional Li‐ion cells which are becoming increasingly critical. The other is the performance requirements of desirable emerging application areas that are beyond the capabilities of traditional LIB technology. Examples include large commercial vehicles,^[^ ^1^ ^]^ high‐altitude long endurance (HALE), high‐altitude pseudosatellites (HAPS), electric vertical take‐off and landing (eVTOL),^[^ ^2^ ^]^ and electric passenger aircraft. The weight of the battery system (BSM) is an especially critical factor for these aviation applications. The battery systems’ performance requirements differ across these applications: power, cycle life, system cost, etc. However, the need for a high gravimetric energy density, 400 Wh kg^−1^ and beyond, is common across them all. Higher energy battery systems will enable these vehicles to achieve extended range, a longer mission duration, lighter vehicle weight, or increased payload. In the following sections, key advantages, limitations, and progress made to extend cycle life, energy, power, and safety of Li–S battery management systems (BMS) are described. Further, recent advances regarding modeling, battery system management, and the integration of Li–S batteries into present as well as future real‐world applications are summarized.

## 2 Lithium–Sulfur Battery Technology

### 2.1 Advantages

LIB systems are the current technology of choice for many applications; however, the achievable specific energy reaches a maximum at around 240–300 Wh kg^−1^ at the cell level.^[^ ^3^ ^]^ Emerging higher‐energy battery systems include advanced Li‐ion technology (e.g., silicon–NMC),^[^ ^4^ ^]^ Li metal–NMC (especially with high‐nickel ternary cathodes),^[^ ^5^ ^]^ Li–S (lithium–sulfur),^[^ ^6^, ^7^ ^]^ and Li–O_2_ (lithium–air).^[^ ^6^ ^]^ In addition to that, solid‐state technology is recently considered as a focus topic in the battery research and industry. As far as exciting and promising these technologies are, the technology readiness level (TRL) should be strongly taken into account when comparing different technologies, as some of them may not be ready for some time yet. According to the EU Integrated Strategic Energy Technology Plan (SET‐Plan) Action 7 for 2030,^[^ ^8^ ^]^ Li–air or rather Li–O_2_ batteries have so far the lowest TRL level. Solid‐state batteries, despite the tremendous attention they have gained, still remain mainly in the laboratory in the form of a small pouch cells at best. A full‐scale solid‐state prototype is being envisaged by Toyota for 2025,^[^ ^9^ ^]^ but Panasonic claims that this technology is more likely to be available in the next decade. First results on Li metal–NMC are very promising,^[^ ^10^ ^]^ but the issue of the availability of the raw materials cobalt and nickel cannot be neglected. Also, in terms of safety, high‐nickel ternary cathodes in combination with lithium still have to be optimized.^[^ ^11^ ^]^

Among these next‐generation battery technologies, Li–S is attracting increasing attention driven by the significant advantages that chemistry can offer combined with the demonstrated technology performance and promising progress made in terms of its TRL in recent years.^[^ ^12^, ^13^, ^14^, ^15^ ^]^

Lithium is the lightest metal and displays a very low standard reduction potential (−3.04 V). These attributes produce an ideal negative electrode which possesses a low operating voltage and high specific capacity. Sulfur is a solid lightweight stable electronegative element that can achieve a high theoretical capacity of 1672 mA h g^−1^ (S) when fully reduced to Li_2_S. When combined in an electrochemical cell with lithium, the formation of one of the highest energy material couples is achieved. Sulfur is also an abundant element which enables the possibility for low‐cost and environmentally compatible battery manufacturing.^[^ ^16^ ^]^ In addition, Li–S technology does not rely on a supply of materials involved in geopolitical or social issues (such as cobalt).^[^ ^17^ ^]^ This factor will become even more important in the nearest future when the demand for energy storage increases exponentially. Li–S technology has also been reported to be more environmentally friendly than commercially available NMC–graphite, when taking into account CO_2_ eq km^−1^ being generated.^[^ ^18^ ^]^ Furthermore, sulfur‐based electrodes can be prepared using water‐based processes, reducing the need of energy‐intense toxic solvents commonly used when processing NMC electrodes. In addition, a dry‐transfer film process without using any solvents has been developed.^[^ ^19^ ^]^

**Figure 1:**
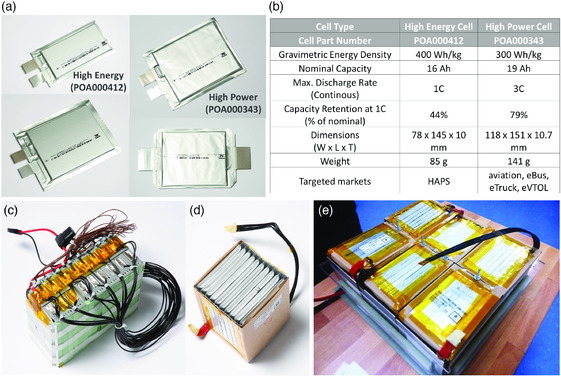
OXIS Energy Li–S pouch cells available in different form factors: a) and more detailed characteristics of high‐energy and high‐power prototypes. b) Representative modules assembled from c) high‐energy and d) high‐power cells along with an example of a e) prototype battery pack.

Today, there are still only very few academic institutions or companies which have demonstrated Li–S battery technology at a TRL greater than 5.^[^ ^20^, ^21^, ^22^, ^23^, ^24^, ^25^, ^26^ ^]^ BASF and SION power worked on Li–S pouch cells^[^ ^27^ ^]^ but have not published any results for several years. LG Chem recently published a press release on a drone powered by Li–S pouch cells with specific energy as high as 410 Wh kg^−1^ and stated that commercial cell production is expected to begin in 2025.^[^ ^28^ ^]^ The drone, called EAV‐3, was codeveloped with Korea Aerospace Research Institute (KARI). A pouch cell with a specific energy as high as 470 Wh kg^−1^ was reported by Beijing Institute of Technology.^[^ ^20^ ^]^ Pacific Northwestern National Laboratory has published work on lithium–sulfur prototype pouch cells to bridge the gap between academic and industrial research.^[^ ^29^ ^]^ Dalian University published Li–S pouch cells with LiNO_3_ free electrolyte, a specific energy of 350 Wh kg^−1^, and specific power of 60 W kg^−1^.^[^ ^24^ ^]^ Tsinghua University in Beijing^[^ ^30^ ^]^ and Gebze Technical University^[^ ^31^ ^]^ have built multilayered pouch cells and investigated the critical parameters for the transfer of research findings from coin to pouch cell levels. OXIS Energy Ltd. is a company dedicated to the development of Li–S battery technology and is currently expanding beyond its pilot‐scale production capability at its facilities in Culham, Oxford, UK.^[^ ^32^ ^]^ At these facilities, high‐capacity (>15 Ah) Li–S pouch cells are routinely produced which exceed 400 Wh kg^−1^ at a TRL/manufacturing readiness level (MRL) of 7–8. The Li–S cells are produced in several form factors, with cell design and components tailored to meet the demands of customers, enabling evaluation of Li–S technology in a wide range of real‐world application areas (**Figure** **1**a,b). OXIS Energy's Li–S technology is under continuous development, enabling the expectation that production of high‐energy Li–S cells of 500–600 Wh kg^−1^ will become possible in the next few years.^[^ ^33^ ^]^ OXIS Energy and CODEMGE recently signed a lease agreement to build the world's first Li–S manufacturing plant.^[^ ^32^ ^]^ In addition, plans by the company Morrow to build lithium–sulfur Gigafactories in Norway are under way.^[^ ^34^ ^]^

### 2.2 Limitations

The main challenges to resolve are cycle life and rate capability. The relatively short cycle life, compared with conventional Li‐ion technology, has its source in the use of a lithium metal‐based negative electrode, especially in combination with highly reactive polysulfides.^[^ ^30^ ^]^ The electrolyte according to the state of the art dissolves a high amount of highly reactive polysulfides that indirectly stresses the anode. It is known that LiNO_3_ in combination with lithium polysulfides plays an important role in passivating the lithium anode.^[^ ^35^, ^36^ ^]^ This depends on sulfur loading in the cathode.^[^ ^37^ ^]^ Below a certain threshold of the concentration of sulfur species, polysulfides can have a beneficial effect. Above a certain sulfur amount, the current density is increased,^[^ ^38^ ^]^ causing dendrite formation, or mossy lithium growth is accelerated.^[^ ^8^, ^23^ ^]^

The development of a stable and reversible lithium metal electrode is of utmost importance for high‐energy battery research,^[^ ^39^, ^40^ ^]^ and it provides the greatest opportunity to improve the performance of Li–S battery technology. It is noteworthy that the generic development of this component is also required for other next‐generation battery systems including Li metal–NMC systems and high‐energy solid‐state battery systems.^[^ ^26^, ^41^ ^]^ Electrolyte depletion, caused by electrolyte consumption at the anode/electrolyte interface, is the major cause of the low cycle life of Li–S technology.^[^ ^42^ ^]^ Improving the cycle life of Li–S battery systems is an important metric for all applications. The rate of electrolyte depletion within Li–S systems drives the need for excess electrolyte and lithium to be added to cells,^[^ ^26^, ^43^, ^44^ ^]^ both of which reduce the gravimetric and volumetric energy density of the system.

Li–S technology has made significant progress in the area of specific energy together with power performance.^[^ ^7^, ^30^, ^45^, ^46^, ^47^ ^]^ However, the limited volumetric energy density resulting from the use of low density and highly porous cathode structures combined with the intrinsic low density of the active material sulfur is still a road block for the implementation of Li–S technology in EV other than trucks and buses. The difference in developing a cell suitable for high‐energy applications compared with a cell designed for high‐power applications includes system design and cell design modification; however, enabling a significant development in performance comes down to the design of the cell components, such as the structure of the cathode and fundamental material properties such as electrolyte system development.^[^ ^29^ ^]^ Within the battery research community, significant efforts have been made to improve the performance of the cell components and materials used within Li–S batteries.^[^ ^25^, ^40^, ^48^, ^49^, ^50^ ^]^ The selected examples are briefly summarized below.

In regards to cathode adaption, a variety of carbons/sulfur composite materials has been synthesized and evaluated over the past decade. The intrinsic carbon porosity has been adapted using various templates and precursors. Also, different carbon morphologies (carbon nanotubes, graphene) have been used.^[^ ^7^, ^45^ ^]^ However, the impact of secondary macroporosity created by the interspace between particles and binders has often been neglected but is about to be addressed in more detail.^[^ ^23^, ^42^, ^51^, ^52^ ^]^

In regards to the lithium anode, promising material concepts such as conductive or in‐conductive frameworks, ionically conductive coatings, spacer concepts, and in situ solid electrolyte interfaces by special electrolyte additives have been developed and analyzed.^[^ ^8^, ^46^, ^53^ ^]^ To bring these material concepts into prototype cells, dead volume and additional inactive material weight or volume need consideration. Coatings should be ionically conductive and maintain a certain mechanical flexibility. Metal–lithium alloys are also an interesting concept but might lower the cell voltage, overall cell energy, and should be stable versus the highly reactive polysulfides.

In terms of electrolytes, ether‐based electrolytes are still promising candidates.^[^ ^54^ ^]^ The concept of sparingly polysulfide solvating electrolytes which intrinsically hamper the polysulfide dissolution and minimize the shuttle effect is promising and allow functioning of the cell without the common LiNO_3_ additive which has been reported to lead to gas formation. However, the mass density and kinetic limitations of these electrolyte systems have to be addressed. Solid electrolytes,^[^ ^55^ ^]^ especially the glass ceramic ones, can also inhibit the polysulfide shuttle. However, the cathode tortuosity and processing have to be strongly adapted as intimate contact between the solid electrolyte and sulfur–carbon composite is crucial.^[^ ^56^ ^]^ Polymeric electrolytes are easier to process but need to be run at elevated temperatures, leading to the partial dissolution of polysulfides and a charge/discharge behavior, which is known from state‐of‐the‐art of ether electrolytes.^[^ ^57^ ^]^

Further developments are, however, still required to enable Li–S technology to fulfil its potential. With respect to the cell‐level limitations, one important consideration is the geometry of commercially available lithium foil (minimum thickness of 50 μm and maximum width of 10 cm^[^ ^58^ ^]^), which limits the overall cell geometry and the optimization of the ratio between the active and inactive component mass. Consequently, nickel tabs have to be adapted for these electrode geometries and for each application (high power vs high energy, see Section 2.3), as these tabs usually play a key role in cell cooling^[^ ^59^ ^]^ as well as transfer current.

Another limitation is the pouch cell as a cell type, as some applications prefer cylindrical cells with a stainless steel casing. As Li–S cells are normally subjected to a drastic volume change, winding and using the lithium anode and sulfur cathodes into rigid cylindrical housings can be detrimental. In addition, steel housing limits the overall energy density.^[^ ^60^ ^]^

To tackle the limitation in terms of volumetric energy, thinner than 50 μm lithium foils are required, ideally without current collectors such as nickel or copper as these have a detrimental impact on the gravimetric energy density. Lithium as an anode is ductile; hence, flexural stiffness is limited as well. A further approach to minimize inactive mass is to use perforated aluminum current collectors on the cathode side. This requires a free‐standing active cathode layer, such as dry film coatings or buckypaper. The scale‐up of these films has definitely improved over the past decade but is still limited when considering Gigafactory scale.

To facilitate focused and high‐value material research, it is suggested that the scientific community should regard a Li–S cell in its entirety and consider the interplay of components and the electrolyte on the cell‐level performance. The possible approaches will be detailed below.^[^ ^15^, ^23^, ^61^ ^]^

### 2.3 Approaches to Improve the Cycle life, Energy, Power, and Safety of Li–S Technology

#### 2.3.1 Cycle Life

**Figure 2:**
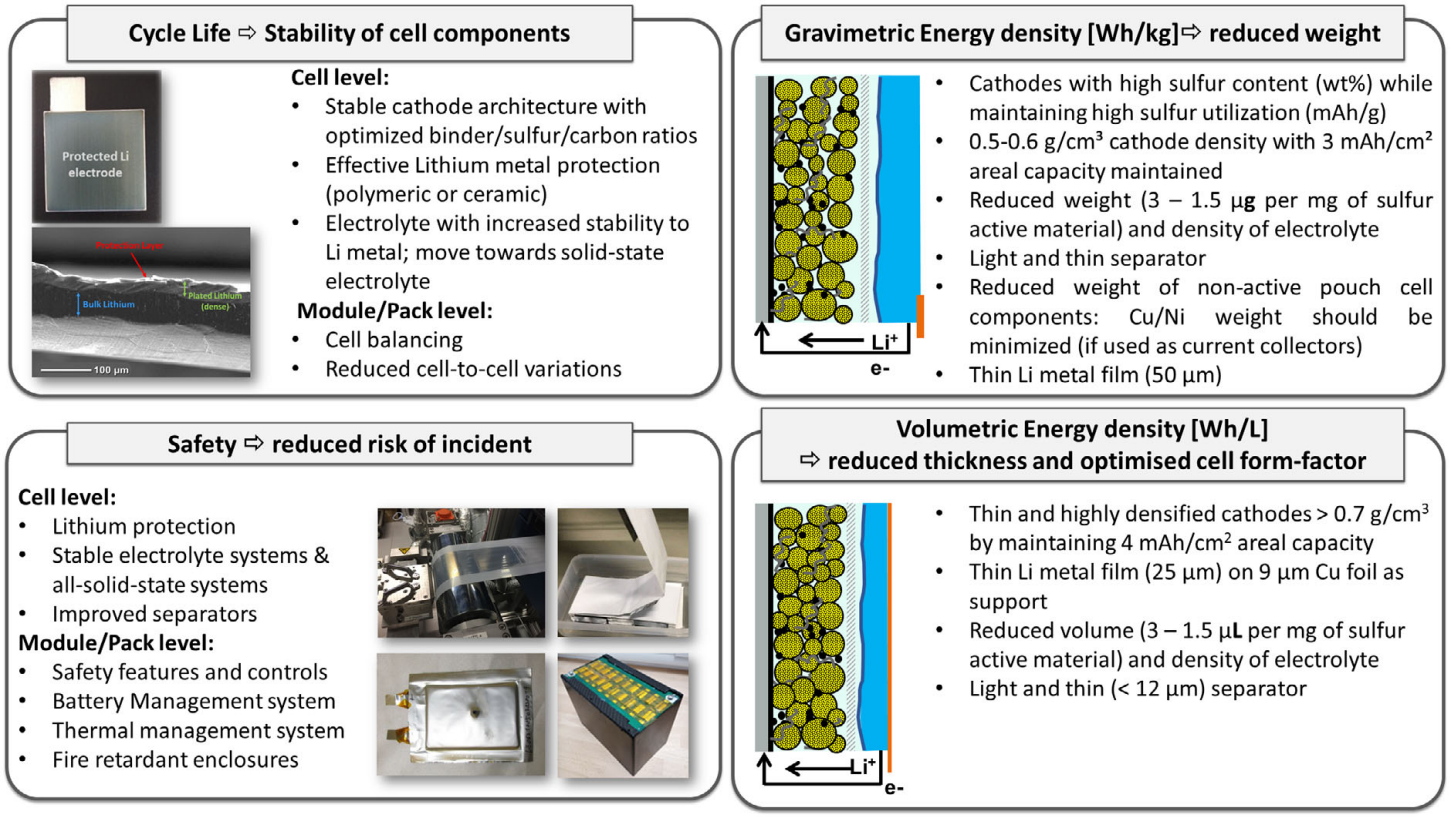
Key factors affecting the main performance characteristics of Li–S pouch cell technology, from materials to system levels.

So far, the most promising approaches to improve the anode/electrolyte interface have been the development of stable electrolyte systems^[^ ^25^, ^62^ ^]^ and the use of solid‐state electrolyte coatings.^[^ ^46^, ^48^, ^63^ ^]^ The realization of a stable lithium anode is crucial to extend the cycle life but it also provides the opportunity for improvements to specific energy, energy density, power performance, and safety. OXIS Energy is actively developing scalable lithium metal protection concepts to stabilize the lithium metal–electrolyte interface within Li–S batteries and enable the isolation of lithium metal from the electrolyte component in prototype cells. An example of a protected lithium electrode produced by OXIS Energy is shown in **Figure** **2**, where the scanning electron microscopy image highlights the ability to plate a dense lithium metal beneath the protection layer. Cell design is a critical parameter for conducting material‐level research into lithium‐based anodes; the use of small pouch cells has been of significant benefit to enable the use of realistic electrolyte volumes and stack pressure. For electrochemical testing, especially of lithium half cells, a minimum charge passed per step of 3 mA h cm^−2^, with a minimum current density of 0.3 mA cm^−2^ that should be implemented, and with current densities >1.5 mA cm^−2^ that should be targeted. The electrolyte loading (E/S ratio) should also be minimized and kept below 2.0 μL mAh^−1^ of charge passed per step. Under these conditions the true impact of material‐level developments can be clearly identified.

#### 2.3.2 Energy (Gravimetric vs Volumetric)

A careful and holistic cell design is the key to achieving high values of gravimetric (Wh kg^−1^) and volumetric energy density (Wh L^−1^).^[^ ^7^, ^23^, ^43^, ^45^ ^]^ The energy density of Li–S technology is a key development metric, especially required for applications in which space is limited, such as EVs. There are three main approaches to increase the energy density (Figure 2).

Increasing the cathode density. Due to the low intrinsic density of both carbon and sulfur, the current electrode tap densities range approximately between 0.4 and 0.6 g cm^−3^. Increasing this density values to >0.7 g cm^−3^ while maintaining areal capacities higher than 4 mAh cm^−2^ enable an increase in energy per volume. Cathode densification will however significantly reduce the volume available for the uptake of electrolyte and might kinetically hamper the conversion mechanism, especially in the electrolyte with high lithium polysulfide (LiPS) solubility.^[^ ^23^, ^64^ ^]^ Hence, the cathode density has to be tailored in conjunction with electrolyte development.

Second the electrolyte volume has to be decreased for both gravimetric and volumetric energy densities, so that the conversion mechanism can take place while polysulfide shuttle and electrolyte depletion are minimized. The total electrolyte volume within a cell must be considered and limited.^[^ ^24^, ^25^ ^]^ Electrolyte densities can range from 1 to 1.5 g cm^−3^, depending on the conductive salt concentration^[^ ^65^ ^]^ or if fluorinated solvents^[^ ^25^, ^62^ ^]^ are used. To tackle the issue of a low volumetric energy density (in Wh L^−1^), the mass density of the electrolyte is less important than for gravimetric energy density (in Wh kg^−1^).^[^ ^49^ ^]^ Reducing the content of electrolyte from 3 to 1.5 μL or per mg of S active material^[^ ^24^ ^]^ will decrease the weight of the electrolyte and the free volume required for its uptake. This is of importance, as in all known Li–S cell concepts, the electrolyte may take a large fraction of the cell (>40 % of the cell weight and volume^[^ ^23^, ^52^ ^]^). The strategy on electrolyte development consequently involves 1) development of an electrolyte with low polysulfide (PS) solubility^[^ ^25^, ^49^, ^62^, ^66^ ^]^ or redesign of the cathode/cell and the accompanied process adaption for the use of solid electrolytes. These concepts have the potential to increase the reversibility of the system and sulfur utilization while reducing the required content of the electrolyte as sulfur species mainly exist in the solid state. 2) Development of a new‐generation electrolyte for Li–S cell enabling increased average discharge voltage. 3) The reduction of lithium excess to only 20% and hence, decreasing the thickness of the lithium anode to ≈25 μm (corresponding to 5.15 mAh cm^−2^ usable areal capacity) is another important approach to reach higher values for both energy per mass and energy per volume. This can be done by further developing new coating techniques for the application of thin lithium metal films, such as melt processing or physical vapor deposition (PVD).

#### 2.3.3 Power

Only a few studies investigate power capability from a holistic point of view at the pouch cell level. It is widely accepted that Li–S technology is not going to compete with the most powerful Li‐ion cells (with LTO or LFP chemistries, capable of cycling at very high C rates). Nevertheless, the specific power (W kg^−1^) obtained from a carefully designed OXIS Energy Li–S pouch cell^[^ ^67^ ^]^ dedicated for power applications can be as high as 800 W kg^−1^ (for continuous discharge) or reach up to 1500 W kg^−1^ at the peak (10 sec discharge at 90% state of charge [SoC]). A specific discharge peak power is strongly dependent on the SoC% and that is closely related with internal chemistry/electrochemistry taking place in the cell while cycling, and it will be explained in more detail further.

**Figure 3:**
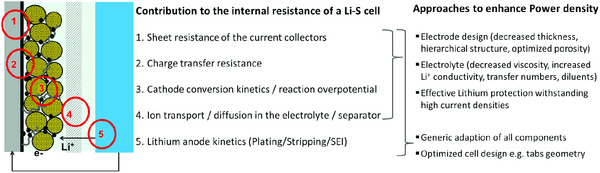
Illustration of which Li–S cell components the internal resistance should be reduced to achieve higher‐power densities.

To increase the power density of a Li–S pouch cell, several components contribute to the internal resistance of a Li–S cell and have to be adapted (**Figure** **3**): thinner electrodes reduce the length of lithium‐ion transport, an important kinetic factor. In addition, similar to LIBs,^[^ ^68^ ^]^ microscale structuring or porosity in the cathode layer can be beneficial for higher‐power systems. Moreover, carbon additives offering a percolating network of electronic pathways increase power capability.^[^ ^42^, ^69^ ^]^ Importantly, electrolyte viscosity and Li^+^ transfer numbers^[^ ^70^ ^]^ are crucial parameters that need further development.

In contrast to LIBs, at medium SoC, the ether‐based standard electrolyte for Li–S batteries changes to a highly viscous gel‐like state, caused by 1) the increase in Li–polysulfide (LiPS) concentration from Li_2_S_8_ to 2 Li_2_S_4_ and 2) the aggregation of lithium polysulfides of stoichiometry Li_2_S_4_ to form dimers and clusters.^[^ ^71^ ^]^ As a result, the electrolyte resistance increases, and thus, the power density decreases. If the electrolyte content is very low, the formation of sparingly soluble LiPS solvate complexes also sets in. These clog the porosity of the cathode and thus strongly impair the ion transport as well as the power density. Furthermore, the deposition or conversion of the charge or discharge products (S_8_ or Li_2_S) is kinetically inhibited.^[^ ^72^ ^]^ It is known that a certain amount of polysulfides is necessary to chemically “activate” the discharge product Li_2_S. However, the high solubility of polysulfides in the electrolyte also produces the so‐called polysulfide shuttle, leading to reduced charging efficiency.^[^ ^73^, ^74^ ^]^ First, promising approaches describe the suppression of polysulfide solubility in the electrolyte by the so‐called “solvent‐in‐salt” concept.^[^ ^75^ ^]^ To improve the insufficient ion transport capacity of the ether/Li‐salt complexes, low‐viscosity hydrofluoroethers (HFE) were investigated as cosolvents or diluents, as they interact very little with the Li ions.^[^ ^76^ ^]^ The low solubility and mobility of LiPS in such systems pose new challenges, as the conversion of the sulfur species is now bound to the surface of the porous cathode structure and does not occur rapidly in the liquid phase. Therefore, the kinetics of the reactions taking place at the phase boundary carbon–sulfur/Li_2_S–electrolyte have to be understood and specifically optimized for the application as high‐performance battery. For example, it has been shown that the saturation of Li_2_S_6_ in the electrolyte can be drastically reduced compared with the reference system (DME/DOL) using a sulfolane/fluoroether based‐solvent system and a low conducting salt concentration of 1.5 M. The electrolyte system has been successfully transferred from coin cells to prototype cells and demonstrated over 200 stable cycles at only 3.5 μL electrolyte per mg sulfur.^[^ ^25^ ^]^

It should be mentioned that higher areal currents generally lead to higher dendrite formation for unprotected lithium.^[^ ^26^, ^77^ ^]^ Consequently, a protected lithium electrode that can operate at high power conditions can significantly alter the cell design. From a prototype pouch cell point of view, tab geometry may also play an important role. In addition, packaging and sealing need special consideration in regard to their use in space and maritime environments.

#### 2.3.4 Safety

OXIS Energy Li–S cell technology has been demonstrated to display superior performance to that of traditional of lithium‐ion technology under a number of safety tests, including nail penetration.^[^ ^78^ ^]^ However, prototype cells recently produced by Fraunhofer IWS evaluating the stability of new electrolyte systems have found that specific electrolyte formulations designed to reduce polysulfide solubility can significantly influence the safety characteristics. Safety tests of 5 Ah pouch cells have revealed that thermal stability is deteriorated by the use of the low‐polysulfide‐solubility sulfolane/hydrofluorether‐based electrolyte^[^ ^25^ ^]^ when compared with a traditional DME/DOL‐based electrolyte. Thermal runaway of cells containing this electrolyte are thought to occur due to the direct and highly exothermal reaction between elemental sulfur and lithium. The difference in the safety of the cell type is due to the fact that less polysulfides emerge from the cathode, and these are polysulfide species that are crucial to passivate metallic lithium and prevent direct contact between sulfur and lithium.^[^ ^37^ ^]^ Above a critical temperature of 125 °C, continuous self‐heating may occur in cells containing this low‐polysulfide‐solubility electrolyte system. However, the development of a Nafion‐coated separator concept^[^ ^79^ ^]^ has been identified as a solution approach and as an additional safety component. In this way, a closed Nafion layer applied on a Al_2_O_3_/polyolefin hybrid separator can prevent contact between the molten sulfur and the metallic lithium and thereby increase the safety of emerging Li–S cell concepts using low‐polysulfide‐solubility electrolytes (Figure 2).^[^ ^80^ ^]^

## 3 Integration of Li–S Cell Technology

In general, the integration of new battery technology to real‐world applications requires significant development from the cell level up to module, pack, and control systems according to the so‐called validation and verification model (V & V model).^[^ ^81^ ^]^ It means that the requirements at, e.g., the aircraft level translate into system definitions at the (sub‐)component and prototype level. In addition, a strategy and concept for the integration of the cells or rather cell packs have to be developed. The integrated cells/cell packs are then evaluated in functional tests, and certification plus safety assessments are conducted to validate the developed concept and strategy.

Significant progress has been made in this direction for Li–S cell technology, and OXIS Energy has integrated its pouch cells into modules and demonstrator battery packs for evaluation in real‐world application scenarios (Figure 1c–e). The development of Li–S modules and battery packs, designed to meet the power requirement profile of a specific application, the so‐called mission profile, occurs through a series of stages from cell and system modeling up to bench testing under simulated conditions. OXIS Energy and its partners have developed advanced BMS, incorporating the most advanced state of health (SoH) and SoC estimators^[^ ^74^, ^82^ ^]^ for Li–S battery systems, a critical requirement for the integration of Li–S battery technology into applications.^[^ ^83^ ^]^

Generally, Li–S technology has received increasing levels of research and development with efforts focused on performance aspects including cycle life, power performance, volumetric energy density, and safety.^[^ ^25^, ^36^, ^42^, ^49^, ^51^, ^62^, ^63^, ^64^, ^65^, ^66^, ^84^ ^]^ To meet the varying performance requirements of emerging applications, OXIS Energy has developed two cell product streams, each with optimized performance characteristics (Figure 1a,b). Application requirements can generally be divided into two sectors: 1) high energy focused with low power requirements (high energy) and 2) moderate energy with the capability for sustained high power (high power).

## 4 State of the Art and Recent Advances of Li–S Cell Modeling for State Estimation

**Figure 4:**
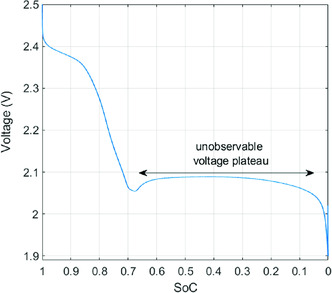
Flat voltage encountered in discharge. This is an example of one of the key differences between many present‐day technologies and Li–S. It is hard to determine SoC from voltage alone because this curve is relatively flat.

Modeling for the purpose of battery management and state estimation has particular requirements in terms of execution speed and computational complexity.^[^ ^85^ ^]^ The modeling techniques which have been developed for LIBs are not applicable for the explanation of discharge phenomena in Li–S cells. The flat open‐circuit voltage curve of the Li–S battery (shown in **Figure** **4**) is a unique characteristic that demonstrates that there is a problem in observing SoC from voltage calculations alone.^[^ ^86^, ^87^ ^]^ The complex electrochemical pathways which exist in the Li–S cell mean that short‐term capacity can vary, so “Coulomb counting” is also ineffective for this particular cell chemistry. In response to the aforementioned problem, two families of estimation techniques have been proposed for Li–S cells in the literature: 1) techniques derived from control and estimation theory, based on nonlinear variants of the Kalman filter,^[^ ^82^, ^86^ ^]^ and 2) techniques that come from computer science such as Adaptive Neuro‐Fuzzy Inference Systems (ANFIS)^[^ ^87^ ^]^ and Long Short‐Term Memory Recurrent Neural Networks (LSTM RNN).^[^ ^88^ ^]^

Although most of the research published in the literature are focused on Li–S SoC estimation, the Li–S cell SoH estimation techniques are also under development from both control theory and computer science. Examples are the general framework describing Li–S cell SoH in terms of capacity fade and resistance growth which have been presented in a study by Wild et al.^[^ ^7^ ^]^ and the SoH estimation technique presented in a study by Knap.^[^ ^89^ ^]^ Looking at the literature, Li–S cell degradation mechanism has been investigated by electrochemists in a number of studies; however, the literature suffers from lack of studies where Li–S cell SoH estimation is investigated for BMS application. Although an insight into understanding the degradation mechanism in Li–S cells using electrochemical models is quite helpful, those models are hardly useable in real‐time applications mainly due to their complexity. In online applications, quick models/estimators are required to generate “good enough” results by providing a proper trade‐off between accuracy and speed.^[^ ^90^ ^]^ In fact, many details related to electrochemical reactions taking place inside a cell are not required to be analyzed in a real‐time application. A couple of studies in the literature where Li–S cell degradation has been investigated by considering the practical application limitations are presented in studies by Knap et al.^[^ ^91^, ^92^ ^]^

All the aforementioned online state estimation techniques (both SoC and SoH) rely on equivalent circuit network (ECN) models. ECN model parameterization for a Li–S cell was conducted in studies by Fotouhi et al.^[^ ^83^ ^]^ and Propp et al.^[^ ^93^ ^]^ for the first time. As the aim of modeling in those studies was to implement the model in a real‐time BMS, quick identification techniques were applied to extract the ECN model parameters. The identification results are then used for Li–S cell state estimation. For example, in a study by Fotouhi et al.^[^ ^87^ ^]^ the simplest form of an electric circuit battery model (i.e., internal resistance model) is parameterized and its parameters are used for SoC estimation. In that study, three inputs including open‐circuit voltage (*V*_oc_), ohmic resistance (*R*_O_), and the derivative of resistance with respect to SoC (d*R*_o_ */*dSoC) are used for SoC estimation using ANFIS method. As another example in the study by Propp et al.,^[^ ^86^ ^]^ the Thevenin ECN model is parameterized and used in Kalman‐variant estimators for Li–S cell SoC estimation.

## 5 Current and Future Applications for Li–S Battery Technology

**Figure 5:**
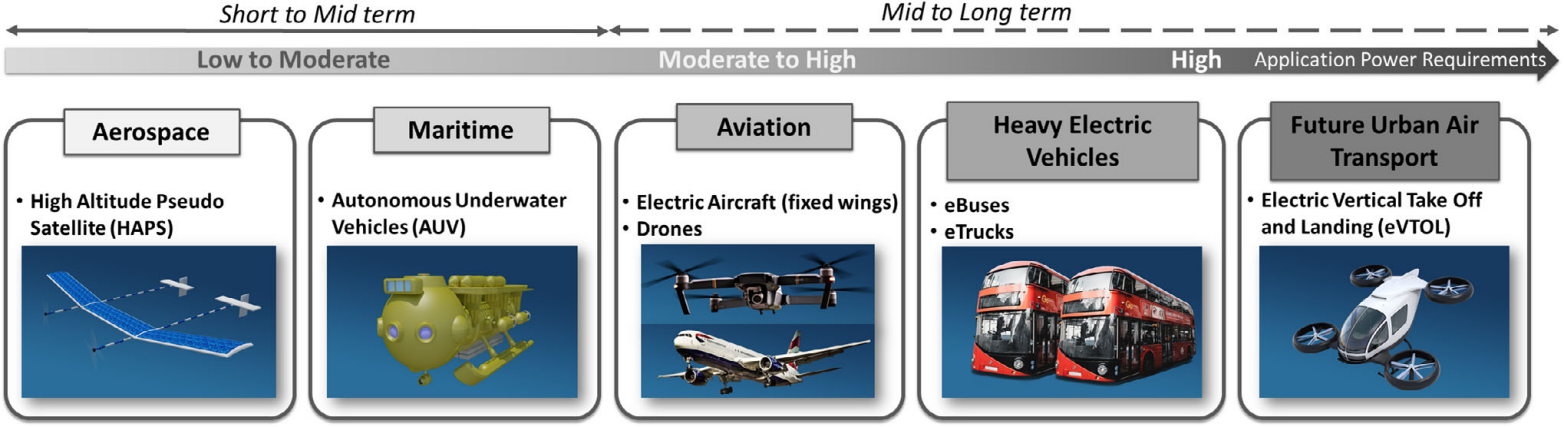
Illustrative schematic demonstrating main markets suitable for Li–S technology now and in the future.

Among the future applications requiring high‐specific‐energy battery systems, a few examples are shown in **Figure** **5** and **Table** **1**, where Li–S technology has the potential to play a significant role in enabling these applications to be successful.^[^ ^14^ ^]^ Principally, the applications can be divided into few segments strongly depending on technology adaption timing and power requirements.

**Table 1:** Illustrative schematic demonstrating main markets suitable for Li–S technology now and in the future with the required values for specific energy, C rate, cycle life, environment, and remaining challenges

Future commercial vehicles^[^ ^21^ ^]^	Aerospace	Maritime	Aviation	Heavy EVs	Future urban air transport
Examples	HAPS	AUVs2	Electric aircrafts (fixed wing) drones	eBuses, eTrucks 4	eVTOL
Required *E*_grav_	>400 Wh kg^−1^	>400 Wh kg^−1^	>300 Wh kg^−1^	>400 Wh kg^−1^	>400 Wh kg^−1^
Required continuous discharge rate	< *C*/5	≈C/10–1 °C	Peak discharge at ≈1–2 °C	Peak discharge at ≈1–2 °C	4–5 °C for take‐off/landing, ≈1–2 °C during cruise
Cycle life	60–200 cycles	60–200 cycles	200–500 cycles	1000 cycles	500 cycles
Environmental requirements	10–40 °C Low pressure (50 mbar)	Low temperature (4 °C) High pressure (45 MPa)	−10–60 °C	−10–60 °C	−10–60 °C
Main remaining challenges	‐	‐	Cycle life (>500) Safety regulations	Cycle life (>1000) Safety regulations	Fast discharge (4–5 °C) while retaining 400 Wh kg^−1^ Fast charge (1–2 °C) Cycle life (>500) Safety regulations. Enhanced thermal management.

### 5.1 Current and Future Application Demands

The current performance of Li–S cell technology is already sufficient for a number of emerging applications which require relatively low power and limited cycle life. There are a number of applications which, apart from demanding high specific energy, also have an increased demand for power. The power requirement is specific to the application and can depend on many factors such as system‐level requirements, battery configuration, energy of the battery pack, usage of the vehicle, etc. A number of key application areas for future battery technologies are discussed in further subsections, starting with applications whose requirements are close to be fulfilled by the current Li–S technology and its TRL/MRL levels, and moving further, the applications which require further development of Li–S technology (in terms of power, safety, etc.) are described. The latter is expected to incorporate Li–S batteries in future. Generally, for the implementation of batteries in drones or aircrafts, the design of the drone, the space for take‐off and landing, and the respective flight modes have to be addressed. As for fixed‐wing aircrafts/drones, more space for take‐off and landing is required and the power requirements are lower compared with rotor blade‐based drones. The latter requires higher power but needs less space for take‐off/landing or rather hovering. The so‐called tilt‐rotor blade allows both flight modes in one drone.^[^ ^94^ ^]^

#### 5.1.1 Aerospace

A growing number of organizations around the world are developing HAPS/HALE aircrafts, with a number of systems having already completed successful test flights. This emerging application requires high‐specific‐energy batteries to enable the maintenance of high‐altitude flights at mid‐high latitudes. HAPS aircrafts are designed to circle in the stratosphere, ≈20 km above the ground, in contrast to satellites, i.e., geostationary Earth orbit (GEO) satellites, which are in an orbit about 36 000 km away from the earth, and low Earth orbit (LEO) satellites, which are about 1200 km away.^[^ ^95^ ^]^ Thanks to that, the launch and maintenance costs are much lower. The stratosphere offers mild weather conditions with little change in wind speed, which result in a stable flight. In addition, due to closer proximity to the Earth (compared with satellites) and greater compatibility with drones and other aircrafts in the stratosphere, it can be greatly beneficial for the next‐generation telecom system.^[^ ^96^ ^]^

For this application, it has been suggested by HAPS vehicle designers that a high specific energy (>400 Wh kg^−1^), low‐to‐moderate charge and discharge rates (<C/5), and low‐to‐moderate cycle lives (60–400) are required. The pack also requires low‐pressure tolerance (≈50 mbar), given its high‐altitude environment.^[^ ^7^ ^]^ Significant progress has been made in the development of Li–S battery systems for HAPS/HALE applications, the Airbus Zephyr 7 aircraft utilized Li–S batteries produced by Sion power. Recently Airbus has announced that it is utilizing Amprius’ silicon nanowire anode LIB technology for its Zephyr S and T models.^[^ ^97^ ^]^ OXIS Energy is currently integrating its Li–S technology into HAPS vehicles. To maximize the benefit of high‐specific‐energy cells, the design of performant but lightweight pack enclosures and control systems must also be considered. OXIS Energy recently developed a high‐energy HAPS module at 380 Wh kg^−1^, which achieves a 95% specific energy retention when moving from the cell to module level.

#### 5.1.2 Maritime

Autonomous underwater vehicles (AUVs) are a growing market and application area for high‐energy battery systems. AUVs are self‐propelled, unmanned, underwater vehicles and can be used for different purposes, such as survey platforms to map the seafloor and observe oceanographic fields, to name few.^[^ ^98^ ^]^ The key requirements of this application are well aligned with Li–S technology today, high specific energy (>400 Wh kg^−1^) combined with low‐to‐moderate power requirements. The battery system must also operate at low temperatures (4 °C) and has to be adapted to withstand high pressures (45 MPa eq. to 6000 m in depth). AUVs usually aim to achieve neutral buoyancy; a recent study has identified that significant benefits to the overall system‐level performance can be achieved via the use of Li–S battery technology.^[^ ^99^ ^]^

#### 5.1.3 Aviation

Depending on the drone, during take‐off and hovering, high‐power density is required. However, relatively low or moderate power is needed during cruise. The mission profile and system‐level requirements for flying applications vary significantly depending on the mission distance and altitude. Hence, mission profile‐specific testing of cells and battery systems must be conducted to gain valuable insights into system‐level performance. Generally, high specific energies (>300 Wh kg^−1^) and moderate power requirements (peak discharge at 1–2 °C) are needed. Hybrid battery concepts comprising both high power and high energy battery are possible.^[^ ^100^ ^]^ Li–S technology may be incorporated into concepts in which lithium–polymer batteries are used for take‐off and the hover mode and a Li–S battery operates as a range extender.^[^ ^101^ ^]^

#### 5.1.4 Heavy EVs

The major benefit of the use of Li–S technology for future eTrucks and eBuses applications is the ability for much lighter battery packs. Reduced battery weight can enable both extended range and increase payload, enabling greater distances between charging, especially important for locations where the installation of significant charging infrastructure may not be viable. Future long‐range/high‐payload eBuses and eTrucks will have similar performance demands, a high specific energy (>400 Wh kg^−1^) at moderate continuous discharge C rates 0.5–0.2 °C and pulses of power in the range of 1–2 °C. Energy density (Wh L^−1^) is less important for this type of EVs when compared with common passenger cars.^[^ ^102^, ^103^, ^104^, ^105^ ^]^ OXIS Energy has conducted mission profile specific testing of its cells and battery systems to gain valuable insights into the system‐level performance of Li–S batteries for eBuses, with more details presented in section 5.2.

#### 5.1.5 Future Urban Air Transport

At the extreme end of energy and power requirements lies the eVTOL aircraft application, an application which demands >400 Wh kg^−1^ at sustained discharge rates of around 1–2 °C along with peak power requirements of up to 4–5 °C.^[^ ^2^ ^]^ With the development of high energy and high‐power battery systems for this urban, manned application, safety is of critical importance. The development of Li–S cell technology to meet the demands of this future application sector is a key area for OXIS Energy.

### 5.2 Application Case Study: Li–S Battery for an Electric City Bus

Various works^[^ ^106^ ^]^ have been conducted regarding the modeling of the Li–S cell chemistry in the past years. A continuous shuttle current measurement method for lithium–sulfur cells was developed by TU Munich in collaboration with Daimler AG in 2020.^[^ ^107^ ^]^ A simple analytical model of capacity fading for lithium–sulfur cells was published by Brno University of Technology in collaboration with OXIS Energy.^[^ ^108^ ^]^ A 3D image‐based modeling of transport parameters in lithium–sulfur batteries was conducted by UCL.^[^ ^109^ ^]^ The electrochemical impedance spectroscopy‐based electric circuit modeling of lithium–sulfur batteries during discharging was evaluated by Aalbourg University.^[^ ^110^ ^]^

**Figure 6:**
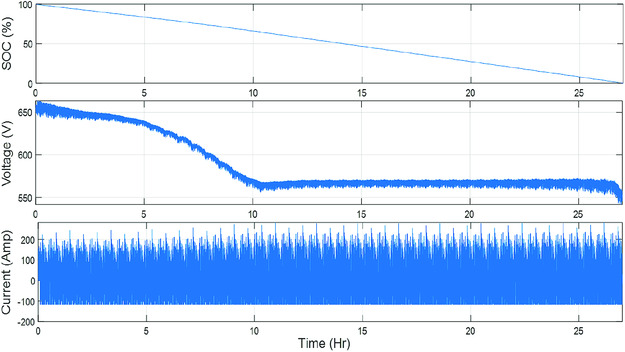
Li–S battery pack SoC, current, and terminal voltage during MLTB simulation case study. Reproduced with permission.^[^ ^111^ ^]^ Copyright 2018, Inderscience.

In a recently reported case study,^[^ ^111^ ^]^ the application of a 19 Ah prototype Li–S pouch cell in an electric city bus was investigated. In that study, a Li–S battery pack was designed as an alternative for the existing LIB pack in a London city bus. Maximum power demand, required energy on board, weight, and other required features of the Li–S battery pack were extracted from the existing electric bus.^[^ ^102^ ^]^ Two existing LIB technologies were considered to be compared with Li–S: 1) LiFePO_4_ and 2) Li_*x*_Ni_*y*_Mn_*z*_CoO_2_.^[^ ^103^ ^]^ Based on the Irizar electric city bus's battery pack specifications,^[^ ^104^ ^]^ there is 282 kWh energy on board when the battery is fully charged. The sizing of the Li–S battery pack was then conducted in a way to have the same amount of energy. Consequently, the number of Li–S cells in series and parallel were calculated and after that, the pack was simulated to investigate its performance in such an application. Millbrook London Transport Bus (MLTB) cycle^[^ ^105^ ^]^ was used in simulations as a standard test procedure. **Figure** **6** shows a case study where the proposed Li–S battery pack was simulated in an electric bus. In that figure, battery SoC, current, and terminal voltage are shown during MLTB simulation.^[^ ^111^ ^]^

**Figure 7:**
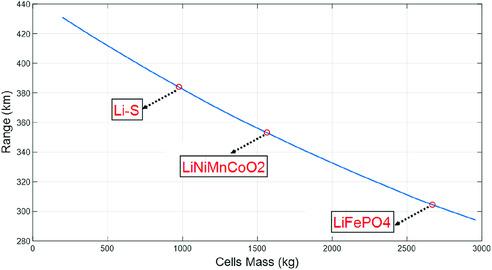
Range of an electric city bus over repeating MLTB cycles using different battery technologies—all battery packs have the same amount of energy (kWh) but they are different in weight depending on the cell's energy density. Reproduced with permission.^[^ ^111^ ^]^ Copyright 2018, Inderscience.

**Figure** **7** shows the range of an electric city bus over the MLTB cycle^[^ ^105^ ^]^ using different battery technologies. In that figure, all battery packs had the same amount of energy (kWh) but they were different in weight depending on the cell's energy density. As shown in Figure 7, the EV range will increase remarkably when Li–S battery technology is used instead of the existing LIB technologies just because of battery lightweighting.^[^ ^111^ ^]^ This result can become even better because the Li–S prototype cell that was used herein had only a moderate energy density of 290 Wh kg^−1^,whereas this number is expected to increase to 400–600 Wh kg^−1^ in the next generations of Li–S cells.

**Figure 8:**
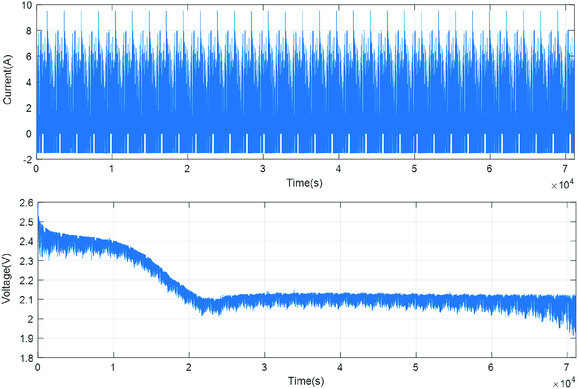
Current profile and cell's terminal voltage measurement during the MLTB test, conducted on a 19 Ah Li–S cell. Reproduced with permission.^[^ ^111^ ^]^ Copyright 2018, Inderscience.

The results presented in a study by Serra^[^ ^111^ ^]^ is not just about simulation; a 19 Ah Li–S cell was actually tested under the MLTB driving cycle condition. Although the whole Li–S battery pack was not built/tested, scaled‐down tests were conducted on single cells in conditions representing the real‐word driving cycles. **Figure** **8** shows the current profile and cell's terminal voltage measurement during the MLTB test, conducted on a 19 Ah Li–S cell. Regenerative braking was also considered in real tests by applying both charge–discharge current values (the negative current demand in Figure 8 represents regenerative charging).

## 6 Outlook and Conclusion

Looking forward to the evolution of electric powertrains, new generations of battery technologies are currently being developed to meet the requirements of emerging applications in terms of cycle life, safety, power, and scalability. Further material‐level developments are required to realize the full potential of Li–S technology and the academic research community has a key role to play in achieving this. However, material‐level research must be conducted within the context of a viable Li–S cell system. Li–S technology has the potential to offer cell‐level specific energy of up to 600 Wh kg^−1^ and thereby enable key performance benefits such as extended range and payload for emerging applications.^[^ ^1^ ^]^ When these key performance benefits are considered together with low cost, availability of materials, stability of supply chains, and demonstrated high TRL/MRL, it is unsurprising that Li–S battery systems have been identified both by academics and by industry leaders as a key enabling technology for future EV applications.^[^ ^12^, ^15^, ^23^, ^33^ ^]^

## Conflict of Interest

The authors declare no conflict of interest.
